# Multi-scale radiomic analysis of sub-cortical regions in MRI related to autism, gender and age

**DOI:** 10.1038/srep45639

**Published:** 2017-03-31

**Authors:** Ahmad Chaddad, Christian Desrosiers, Matthew Toews

**Affiliations:** 1 Laboratory for Imagery, Vision and Artificial Intelligence, École de Technologie Supérieure, Montréal, Québec, Canada

## Abstract

We propose using multi-scale image textures to investigate links between neuroanatomical regions and clinical variables in MRI. Texture features are derived at multiple scales of resolution based on the Laplacian-of-Gaussian (LoG) filter. Three quantifier functions (Average, Standard Deviation and Entropy) are used to summarize texture statistics within standard, automatically segmented neuroanatomical regions. Significance tests are performed to identify regional texture differences between ASD vs. TDC and male vs. female groups, as well as correlations with age (corrected *p* < 0.05). The open-access brain imaging data exchange (ABIDE) brain MRI dataset is used to evaluate texture features derived from 31 brain regions from 1112 subjects including 573 typically developing control (TDC, 99 females, 474 males) and 539 Autism spectrum disorder (ASD, 65 female and 474 male) subjects. Statistically significant texture differences between ASD vs. TDC groups are identified asymmetrically in the right hippocampus, left choroid-plexus and corpus callosum (CC), and symmetrically in the cerebellar white matter. Sex-related texture differences in TDC subjects are found in primarily in the left amygdala, left cerebellar white matter, and brain stem. Correlations between age and texture in TDC subjects are found in the thalamus-proper, caudate and pallidum, most exhibiting bilateral symmetry.

Autism is a complex developmental disability that often appears during infancy, typically in the first three years of life^1^. It is a spectrum disorder that affects about one in 300 children, with individuals affected differently and to varying degrees^2^. The causes of autism are not yet fully understood, and a combination of developmental, genetic, and environmental factors are believed to be involved^3^,^4^.

With the development of *in-vivo* brain imaging technologies such as Magnetic Resonance Imaging (MRI), significant progress has been made toward understanding the physiological characteristics of Autism Spectrum Disorder (ASD). Morphological analysis methods have identified links between regional image measurements and ASD, shedding some light on the mechanisms of this complex disorder. Such methods typically quantify physiological properties of neuroanatomical structures from image data, e.g. volume, thickness, shape, etc., and then identify regions/features for which these properties exhibit statistically significant differences between subject groups (e.g., normal control and ASD) or correlations with variables of interest (e.g., age).

A number of studies have identified physiological differences between ASD and healthy subjects in several key brain regions, including the putamen^5^, cerebellum^6^,^7^, hippocampus^8^,^9^, amygdala^10^, and corpus callosum^11^. The majority of these studies have focused on regional volume or intensity measurements, however, and have not fully exploited the rich information contained in MRI. A recent body of research, commonly referred to as *radiomic analysis*, focuses on texture and shape features derived from image data, that provide a source of information complementary to traditional voxel-wise or volumetric measurements. In general, radiomic analysis hypothesizes that texture operators tuned to an appropriate spatial extent or scale may provide information regarding the microstructure of the biological tissues observed, a hypothesis closely tied to results from scale-space theory^12^. Texture features can be computed by various means including linear filtering operations (e.g., Laplacian-of-Gaussian (LoG)^13^, wavelets) or gray-level co-occurrence matrices (GLCM)^14^, and offer a means of characterizing localized image variations arising from tissue heterogeneity, boundary smoothness^15^, etc. Such features have been successfully applied in a variety of image analysis contexts, including segmentation and computer-aided diagnosis^16^,^17^,^18^,^19^,^20^,^21^,^22^. In lung and head-and-neck cancer data, radiomic features were shown to have significant prognostic power, their signature relating intra-tumor heterogeneity with gene-expression patterns^23^. The links between tumor phenotype diversity, their heterogeneity at cellular and genetic levels, and imaging features derived from different modalities have been validated in numerous studies^24^,^25^,^26^,^27^,^28^. Radiomic features based on texture have also been used to identify subtle differences between brain tissues in control subjects and those of Alzheimer’s patients, related to cognitive impairment severity^19^,^29^.

The primary contribution of this work is to characterize the link between neuroanatomical regions and ASD based on MRI texture features. We propose a region-based radiomics analysis method, whereby textures are first computed across a range of spatial resolutions via multi-scale LoG filtering, after which distributions of filter responses within neuroanatomical regions are summarized by quantifier functions (average, standard deviation and entropy) and compared across groups. While various neuroimaging studies have investigated multi-scale filter or wavelet responses on a voxel-wise basis^30^, our approach characterizes distributions of these measurements within neuroanatomical regions, thus capturing tissue-wise heterogeneity on a per-region basis. In contrast to voxel-wise analysis which requires accurate image registration and correction for large numbers of multiple comparisons, texture analysis requires only region segmentation and correction for multiple comparisons needs only to account for the numbers of regions and features, which are typically much fewer than the numbers of voxels per image. Moreover, the proposed analysis identifies numerous regions exhibiting significant textural differences between healthy control and ASD groups, several of which are consistent with previous results in the literature. As an additional contribution, we broaden our analysis to find regional texture differences linked with the sex and age of subjects. Once more, our method identifies various significant regions related to sex and age, most of which are consistent with findings in the literature.

To our knowledge, this is the first work to investigate the links between MRI textures in neuroanatomical regions and autism, sex and age. MRI textures represent a source of information that is complementary to traditional voxel-wise or volumetric measurements. Our texture analysis method thus provides an additional means of characterizing group differences or correlations in structural MRI. This may potentially lead to a better understanding of the neuroanatomical substrate of diseases such as ASD and the links between normal neuroanatomy and variables such as gender and age.

The rest of this paper is as follows. A review of related work is first presented in Section 2. We then present our proposed method in Section 3, and apply it on real brain MRI data in Section 4. The main findings of our experiments are then discussed in Section 5. Finally, we conclude by summarizing the contributions of this study and providing potential extension of our work.

## Related Work

Our review of relevant work focuses on studies using imaging techniques to identify brain regions/characteristics related to autism, gender and age.

Starting with work related to ASD, various studies using MR imaging have shown that young children with autism had a significantly larger brain volume compared to normally developing peers^31^,^32^. Studies on autism have also identified volume differences in specific brain structures including the cerebellum^6^,^31^, amygdala^31^, corpus callosum^33^, caudate nucleus^34^, hippocampus^31^,^35^ and putamen^5^,^36^. For example, it was shown that caudate nucleus and pallidus volumes were related to the level of ASD-like symptoms of participants with attention-deficit/hyperactivity disorder, and that the interaction of these two structures was a significant predictor of ASD scores^36^. In some cases, contradictory results have been reported. While^8^ found that autistic children had an increase of hippocampal volume which persisted during adolescence, another study including autistic adolescents and young adults reported a decrease in hippocampal volume^35^. Other investigations, such as ref. 37, have shown no significant differences in hippocampal volume between ASD and control subjects. Recent studies on autism have focused on finding abnormalities related to brain development. In ref. 38, it was found that pre-adolescent children with ASD exhibited a lack of normative age-related cortical thinning, volumetric reduction, and an abnormal age-related increase in cortical gyrification. It has been hypothesized that the abnormal trajectories in brain growth, observed in children with autism, alters patterns of functional and structural connectivity during development^39^.

Morphological differences between male and female brains have been explored, such differences are of interest since the prevalence and symptoms of various disorders are linked with gender^40^. For instance, autism is diagnosed four to five times more frequently in boys than girls^41^,^42^, and multiple sclerosis is four times more frequent in females than males^43^. Likewise, anxiety, depression and eating disorders have a markedly higher incidence in females than male, especially during adolescence^44^,^45^. The incidence of schizophrenia between males and females also differs across the lifespan^46^. In terms of brain development, the growth trajectories of several brain regions have been shown to be linked to the sex of a subject, with some regions developing faster in boys and others in girls^47^,^48^. Various studies have also investigated sexual differences associated with autism. A recent study showed that the cortical volume and ventromedial/orbitofrontal prefrontal gyrification of females is greater than males, in both the ASD and healthy subject groups^49^. In another study, the severity of repetitive/restricted behaviors, often observed in autism, was found to be associated with sexual differences in the gray matter morphometry of motor regions^50^.

A number of studies have focused on morphometric brain changes associated with aging^4^. In refs 51, 52, 53, cross-sectional and longitudinal analyses of brain region volumes revealed that the shrinkage of the hippocampus, the entorhinal cortices, the inferior temporal cortex and the prefrontal white matter increased with age. These studies have also highlighted trends towards age-related atrophy in the amygdala and cingulate gyrus of elderly individuals. Conversely, other investigations found no significant volumetric changes of the temporolimbic and cingulate cortical regions during the aging process^54^,^55^,^56^. A recent study applied voxel-based morphometry to compare the white matter, grey matter and cerebral spinal fluid volumes of ASD males to control male subjects^57^. The results of this analysis have demonstrated highly age-dependent atypical brain morphometry in ASD subjects. Other investigations have reported that neuroanatomical abnormalities in ASD are highly age-dependent^58^,^59^.

The vast majority of morphometric analyses in this review have focused on voxel-wise or volumetric measurements derived from brain MRI data. Texture features provide a complementary basis for analysis by summarizing distributions of localized image measurements, e.g. filter responses, within meaningful image regions. Several studies have begun to investigate texture in brain MRI, for example to identify differences between Alzheimer’s and control groups^22^, to discriminate between ASD and TDC subjects^60^, and to evaluate the survival time of GBM patients^17^,^61^. Texture features can be computed at multiple scales within regions of interest, for example multi-scale textures based on the LoG filter have been proposed for grading cerebral gliomas^16^.

Among the works most closely related to this paper is the approach of Kovalev *et al*.^62^, where texture features were used to measure the effects of gender and age on structural brain asymmetry. In this work, 3D texture features based on extended multi-sort co-occurrence matrices were extracted from rectangular or spherical regions in T1-weighted MRI volumes, and compared across left and right hemispheres. This analysis revealed a greater asymmetry in male brains, most pronounced in the superior temporal gyrus, Heschl’s gyrus, thalamus, and posterior cingulate. Asymmetry was also found to increase with age in various areas of the brain, such as the inferior frontal gyrus, anterior insula, and anterior cingulate. While this work also investigated the link between MRI texture, gender and age, it was limited to lateral asymmetry and did not consider textural differences across gender and age groups. Moreover, texture features were obtained from arbitrarily defined sub-volumes that do not correspond to known neuroanatomical regions. In contrast, our work links texture observations to standard neuroanatomical regions obtained from a parcellation atlas, which provide a more physiologically meaningful basis for analysis in comparison to arbitrarily defined regions.

To our knowledge, no work has yet investigated MRI texture analysis within neuroanatomical regions, obtained from a parcellation atlas, as a basis for studying differences related to autism, sex and age.

## Materials and Methods

In this paper, we propose identifying associations between neuroanatomical regions and variables of autism, sex and age using texture features derived from brain MRI data. In our method, MRI volumes are first registered to an atlas and parcellated into 31 key sub-cortical regions, e.g. via a processing pipeline such as FreeSurfer^63^. While our method could also be used to analyze regions of the cerebral cortex, such regions are typically more prone to registration errors^64^. The thinness of the cortex constitutes another obstacle to our approach, which aggregates texture measurements within a 3D volume.

**Figure 1:**
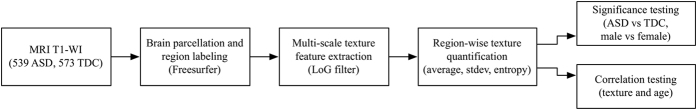
Schematic diagram of the proposed method for identifying significant texture differences related to autism, sex and age, in various brain regions.

Multi-scale texture features are then computed from MRI data by applying a Laplacian-of-Gaussian (LoG) filter across a range of resolution scales, and the distributions of texture filter responses are summarized by the mean, standard deviation and entropy on a per-region basis. Finally, a statistical analysis is performed to identify regions exhibiting significant textural differences between discrete subject groups (e.g. ASD vs. TDC, male vs. female) using permutation tests and Fisher’s method, and correlations with continuous variables (e.g. age) using Spearman rank correlation. An overview of the method is shown in the flowchart of Fig. 1. The following sections describe each component of the method in greater detail.

### Participants and data

T1-weighted MRI data and demographic information for 573 TDC and 539 ASD subjects were obtained from the publicly available ABIDE database (http://fcon_1000.projects.nitrc.org/indi/abide/), an online consortium of MRI and resting-state fMRI data from 17 international sites. In accordance with Health Insurance Portability and Accountability (HIPAA) guidelines, all data are anonymized with no protected health information included. All volumetric images were acquired with a resolution of 1 mm^3^, for a total size of 256 × 256 × 256 voxels.

The demographic and clinical characteristics of subjects in the database are shown in Table 1. A drill-down of this information is also given for the TDC group, which is used in our analyses related to sex and age variables. A number of confounds exist that complicate analysis. Due to the higher incidence of autism in male subjects, data are significantly imbalanced with regard to sex (i.e., 948 males vs. 164 females). Moreover, the database consists primarily of young subjects with developing brains, and inter-subject variations in the shape and size of neuroanatomical regions may impact analysis. Finally, the data is multi-site and thus subject to inter-site variations in MRI intensity. Although various pre-processing steps are applied to normalize the data across sites and subjects, these confounds could influence our analysis and are dealt by performing two types of analyses: a permutation analysis which accounts for inter-subject and inter-site variability in the null distribution, and a bootstrap analysis with multiple balanced data subsets. More details about these analyses are provided in the Statistical Analyses section.

### Image preprocessing and region labelling

**Figure 2:**
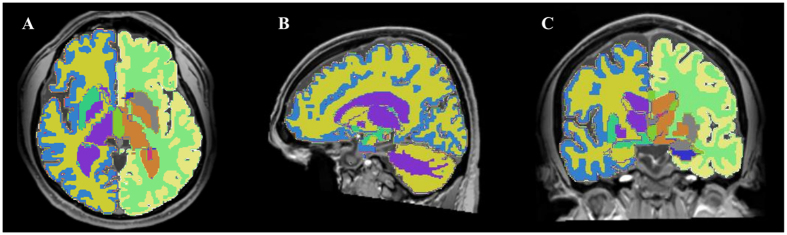
Labeling example of brain regions. (**A**) Axial, (**B**) Sagittal, (**C**) Coronal. Each color represents a different region label. Non-brain tissues (e.g., skull) are shown here for visualization purposes.

We used a preprocessed version of the ABIDE I database, provided by the Preprocessed Connectomes Project (PCP) and publically available for download at http://preprocessed-connectomes-project.org/abide/. T1-weighted MRI images from this database were preprocessed using the *recon-all* pipeline of the FreeSurfer 5.1 tool (http://surfer.nmr.mgh.harvard.edu/)^63^, which involves the following six steps: (1) small-motion correction by averaging the available volumes of subjects, (2) (non-uniform) intensity normalization, (3) affine registration of volumes to the MNI305 atlas, (4) skull-stripping, (5) non-linear registration and further normalization using the Gaussian Classifier Atlas (GCA), and (6) brain parcellation and subcortical region labelling using the GCA. The outputs of this pipeline used in this work are the skull stripped, intensity normalized brain volumes in the subject space (i.e., the *brain.mgz* file), and the sub-cortical labelling of these volumes into 45 hemisphere-distinct regions (i.e., the *aseg.mgz* file). For our analysis, only 31 of the most prominent regions were used. Figure 2 shows an example of brain regions labelled using this pipeline.

**Figure 3:**
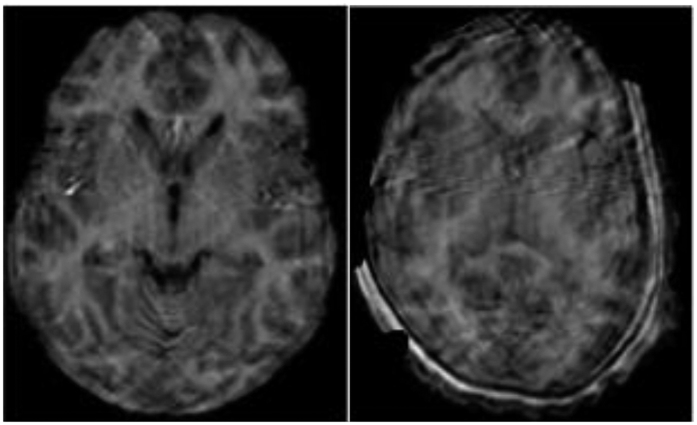
Examples of excluded data.

A subset of ABIDE preprocessed anatomical volumes are manually labeled as quality assessment (QA) failure cases by two raters (rater 2: 52 volumes, rater 3: 108 volumes). While some of these cases correspond to important motion artifacts or segmentation errors, a visual inspection reveals that most of these failures are due to factors that do not affect the subcortical segmentations used in our method (e.g., erroneous segmentation of the cortex or spinal cord). Our primary analysis is thus performed on all but a subset of 8 volumes where QA failures result in noticeable subcortical segmentation errors (e.g., see Fig. 3). These volumes correspond to the following subject IDs: UM_1_0050288, UM_1_0050309, UCLA_1_0051244, UCLA_1_0051245, UCLA_1_0051246, UCLA_1_0051270, UCLA_2_0051296 and UCLA_2_0051310. To assess the impact of excluding larger sets of subjects on our results, we additionally evaluate our method excluding entire sets of volumes flagged as “fail” or “not assessed” by ABIDE raters. We note that virtually identical sets of significant features are identified, however, at higher corrected *p*-values due to fewer data samples. Figures 11 and 12 show results following exclusion of 52 subjects flagged by rater 2.

### Texture feature extraction

**Figure 4:**
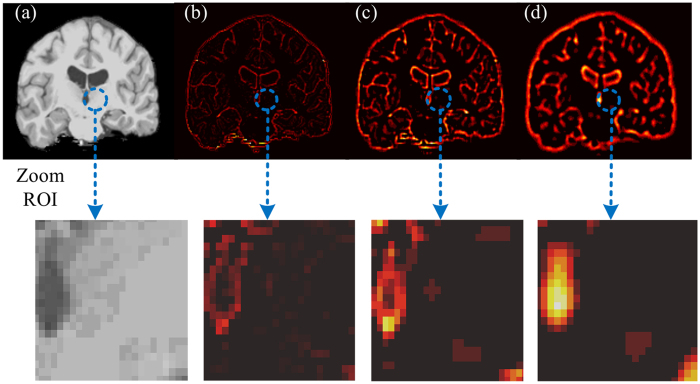
Example of multiscale texture. First row: Texturized T1-WI brain image (**a**) by LoG filter. The corresponding images selectively display, (**b**) fine, (**c**) medium and (**d**) coarse textures. Second row: zoom image illustrates the texture type within an ROI (blue color).

Once the subject volumes have been segmented into a set of common regions, we then extract texture features from each of these regions. Although various texture descriptors have been proposed in the literature, in this work, we adopt texture features based on the multi-scale Laplacian-of-Gaussian (LoG) filter^65^, which has been used successfully in various medical imaging applications^13^,^16^,^66^. Texture feature extraction is a two-step process, involving (1) filtering the image over a range of spatial resolutions, and (2) using a set of quantifier functions to summarize filter responses within regions. We applied the LoG filter at three different scales *σ*, on the intensity normalized T1-weighted images of each subject. The LoG filter is a symmetric 2^nd^ order spatial derivative filter resulting from a Gaussian smoothing operation with a kernel of standard deviation *σ* followed by the Laplacian operator. In this context of this work, it serves as a generic differential operator that responds to local image variations such as edges or blobs within a volume. We hypothesize that filtered volumes encode properties of tissue heterogeneity and local structure that may be connected to the development abnormalities of ASD. Moreover, by varying the scale parameter *σ*, the LoG filter can capture textural image variations potentially arising from tissues and structures across a range of spatial resolutions. In this study, we considered three different scales: (1) *σ* = 0.5 mm (fine texture), (2) *σ* = 1.5 mm (medium texture), and 3) *σ* = 2 mm (coarse texture). Figure 4 shows an example of an image texturized using the LoG filter. The next step of the feature extraction process is to encode texture filter responses within each region. This is done by summarizing distributions of filter responses using three quantifier functions: the average (A) and standard deviation (SD) representing the 1^st^ and 2^nd^ order moments and the entropy (E) representing the distribution uncertainty. Let *f*_*σ*_(*x, y, z*) be the LoG filter response at voxel (*x, y, z*) with scale parameter *σ* ∈ {0.5, 1.5, 2}, and denote as denote as _*i*_ the set of voxels in region *i* ∈ {1, …, 31}. The quantifier functions are defined as follows:


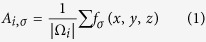



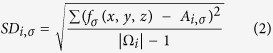



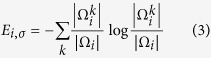


where 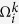 is the subset of voxels within the *k*-th interval of values using a uniform discretization into 256 intervals. In the context of this work, each neuroanatomical region *i* is thus represented by a vector *x*_*i*_ of 9 texture features:


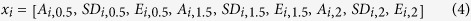


Although the standard deviation and entropy both measure the homogeneity/heterogeneity of filter response, entropy is typically less sensitive to outliers in a region (i.e., voxels with a response significantly different from the region mean) than standard deviation. This is due to the fact that it uses the distribution of voxels within different intervals of filter responses, but not the actual filter response values. Therefore, it may be more robust to errors in the parcellation step than standard deviation.

### Statistical analyses

We performed three statistical analyses to identify brain regions where texture features are informative regarding differences between discrete subject groups (i.e. autism and sex labels) or correlated with continuous variables (i.e. age). To account for multiple comparisons (i.e., 31 sub-cortical regions × 3 filter scales × 3 texture features = 279 tests), all *p*-values obtained from significance testing were simultaneously corrected according to the Holm-Bonferroni method^67^. A threshold of *p* < 0.05 on corrected *p*-values was used to identify statistically significant regions.

### Texture differences related to autism

A major consideration is to account for differences in imaging protocols and demographics across sites. To avoid this problem, we could perform a similar hypothesis test independently for each site and then combine the obtained *p*-values using a standard approach such as Fisher’s method^68^. However, such approach is not feasible due to the data paucity and variability: the number of subjects per site is small and the range of inter-site subject demographic variability is high. Instead, we obtained robust *p*-value estimates using a two-sided permutation test. In this test, we generated 100,000 samples from the null distribution (i.e., same median feature) by randomly permuting the diagnosis group labels of all subjects, and measuring for each sample the absolute difference of median (ABM) between permuted ASD and TDC subjects. We then computed a *p*-value, for each feature, filter scale and region, as the percentage of permutations for which the ABM is greater than the ABM obtained using the true diagnosis group labels. As mentioned above, the 279 *p*-values obtained in this way were corrected using the Holm-Bonferroni procedure. Although the Wilcoxon rank-sum test could also have been used for this analysis, such approach is typically less informative because it measures differences based solely on rank. Moreover, the choice of using the median instead of the mean is justified by the fact that texture features are not necessarily normal distributed.

For the sake of completeness, we also generated 100 balanced bootstrap samples using the true group diagnosis labels, each one containing 100 ASD and 100 TDC subjects. We then obtained a *p*-value for each sample by applying a Wilcoxon rank-sum test, and used Fisher’s method to combine them in a single aggregate *p*-value. Since the *p*-values of different bootstrap samples are correlated, the aggregate values obtained with Fisher’s method are overly optimistic (high chance of false positives) and cannot be used directly. However, these values can be compared relative to one another to identify potential regions of interest.

### Texture differences related to sex

A similar analysis was conducted to identify brain regions exhibiting significant texture differences between healthy (i.e., TDC) male and female subjects. A permutation test was first employed to compare the absolute difference of median feature values between male and female subject groups, with those obtained from 100,000 random permutations of sex labels (i.e., null distribution). Once again, *p*-values were estimated as the ratio of permutations leading to a higher absolute difference of median than those obtained with the true sex labels, and were corrected using the Holm-Bonferroni method. Moreover, a probability test based on Fisher’s method was used to combine the *p*-values of 100 balanced bootstrap samples of 50 TDC males and 50 TDC females into a single aggregate *p*-value.

### Texture differences related to age

Finally, the last analysis identified brain regions for which textures are significantly correlated with age. For this analysis, we first computed the Spearman rank correlation coefficient (SRCC) between the age of TDC subjects and their texture features of each brain region, and considered brain regions having a moderate to high correlation value, i.e. *ρ* > 0.4. To further validate the results of this analysis, we also conducted two additional tests. In the first test, we generated 100,000 samples from the null distribution (i.e., features and age are uncorrelated) by randomly permuting the subjects’ age values, and computing the SRCC of these permutations. We obtained *p*-value as the percentage of permutations having an absolute correlation greater than the true absolute correlation. Once more, these *p*-values were then corrected using the Holm-Bonferroni procedure. In the second test, we generated 100 bootstrap samples of 100 TDC subjects (both male and female) and computed the SRCC for each of these samples. We then obtained a lower-bound on the 95% confidence interval (i.e., the highest SRCC value, smaller than 95% of sampled SRCC values), which we use to further validate the significance of correlation values.

## Results

In this section, we present the results for three proposed analyses: texture differences between subject groups (i.e. autism and sex), and texture correlations with continuous variables (i.e. age).

### Texture differences related to autism

**Figure 5:**
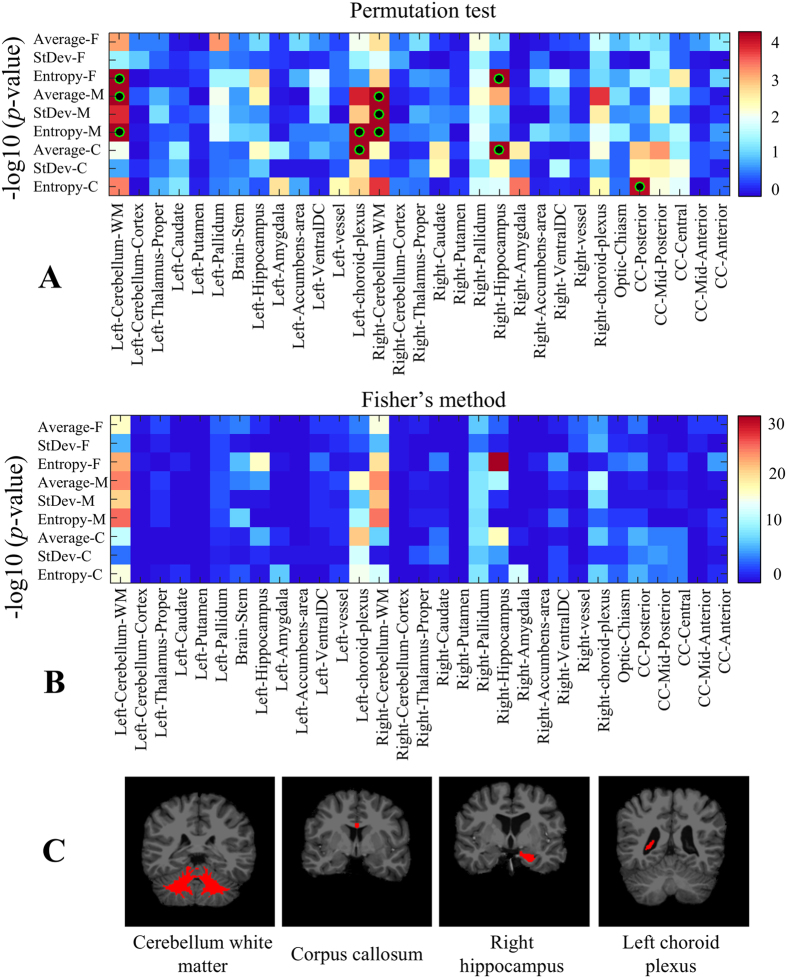
Region-wise texture feature differences between ASD and TDC subjects. (**A**) Heatmap of uncorrected *p*-values (−log10 space), based on 100,000 permutations of diagnosis group labels. Green-black circles indicate region-scale values with significant *p*-values following Holm-Bonferroni correction. (**B**) Heatmap of Fisher’s method *p*-values (−log10 space), based on 100 bootstrap samples of 100 ASD vs 100 TDC subjects. (**C**) Regions showing significant texture differences with corrected *p* < 0.05.

The heatmap of uncorrected *p*-values (in -log_10_ space) obtained from the permutation test is depicted in Fig. 5A. Regions that are significant after Holm-Bonferroni correction, with a confidence level of 0.05, are identified with a green-black circle. We notice several brain regions with significant differences in texture features between ASD and TDC subjects: left and right cerebellar white matter, left choroid-plexus, right hippocampus and posterior of corpus callosum. Table 2 gives the detailed corrected *p*-values of these regions, which are depicted in Fig. 5C. To analyze properties related to symmetry and scale, *p*-values are reported for both brain hemisphere, when applicable, as well as all three texture scales (i.e., fine, medium, coarse).

We observe a pronounced bilateral asymmetry for the choroid plexus and hippocampus regions, as well as a notable symmetry for the cerebellar white matter. Moreover, we see that the scale of significant features varies from one region to another, here with fine-scale features being more relevant for small or narrow regions like the hippocampus, and coarser ones for larger regions like the corpus callosum. This indicates the usefulness of multi-scale features in our analysis. With respect to quantifier functions, the average and entropy functions are more relevant than the standard deviation. As previously mentioned, this could be due to the higher sensitivity of the standard deviation to region boundaries obtained from parcellation.

The *p*-values obtained via Fisher’s method are shown in Fig. 5B. We observe the highest *p*-values for left and right cerebellar white matter, left choroid-plexus, right hippocampus and right pallidum. These results further validate the set of significant regions identified on the permutation test, in addition to their patterns of bilateral symmetry and texture scale.

### Texture differences related to sex

**Figure 6:**
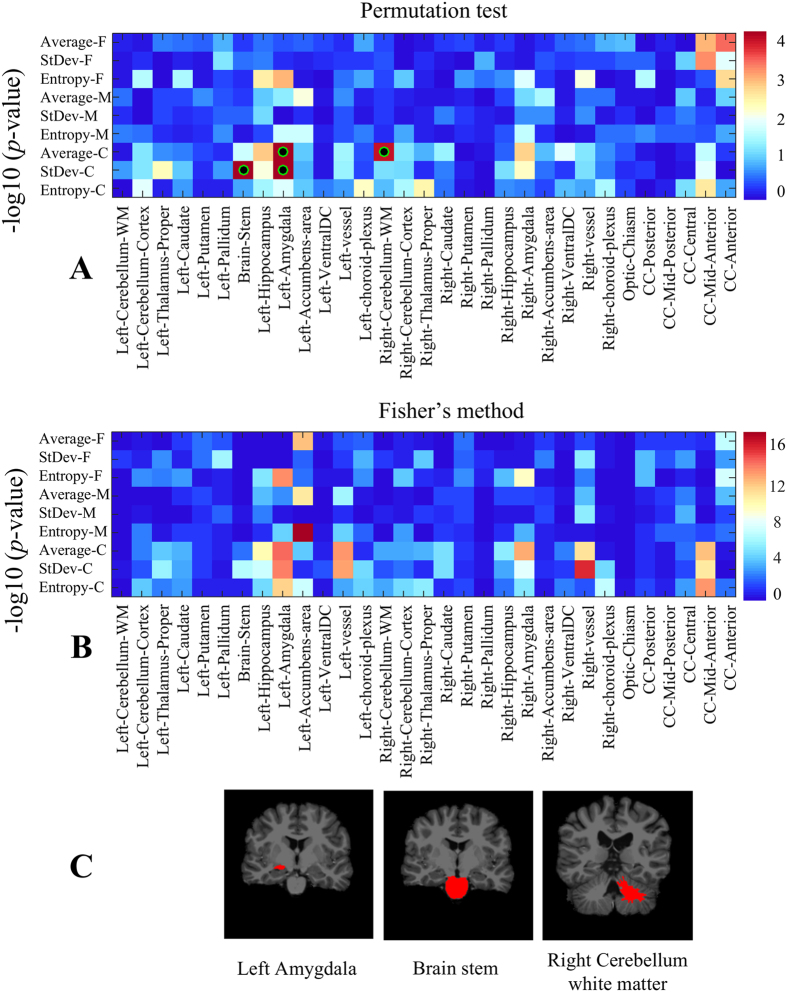
Region-wise texture feature differences between male and female TDC subjects. (**A**) Heatmap of uncorrected *p*-values (-log_10_ space) based on 100,000 permutations of sex labels. Green-black circles indicate region-scale values with significant *p*-values following Holm-Bonferroni correction. (**B**) Heatmap of Fisher’s method *p*-values (−log_10_ space), based on 100 bootstrap samples of 50 males vs 50 females. (**C**) Regions showing significant texture differences with corrected *p* < 0.05.

As in the previous analysis, we summarize the results of the permutation test on sex-related texture differences using a heatmap of uncorrected *p*-values (Fig. 6A). Three regions, namely, brain-stem, left amygdala, and right cerebellar white matter (Fig. 6C), exhibit significant texture differences, with *p* < 0.05 following Holm-Bonferroni correction (see detailed corrected *p*-values in Table 3). The link between these regions’ texture and sex is further demonstrated by the results of Fisher’s method (Fig. 6B). Based on these results, we see a skew toward coarse texture features in regions exhibiting the strongest differences, suggesting that texture differences between male and female subjects occur at a greater structural scale. We also observe that differences are more pronounced in the left hemisphere, in particular for the amygdala. Although not fully understood, these structures are known to exhibit functional lateralization (see the Discussion for details).

### Texture correlations with age

**Figure 7:**
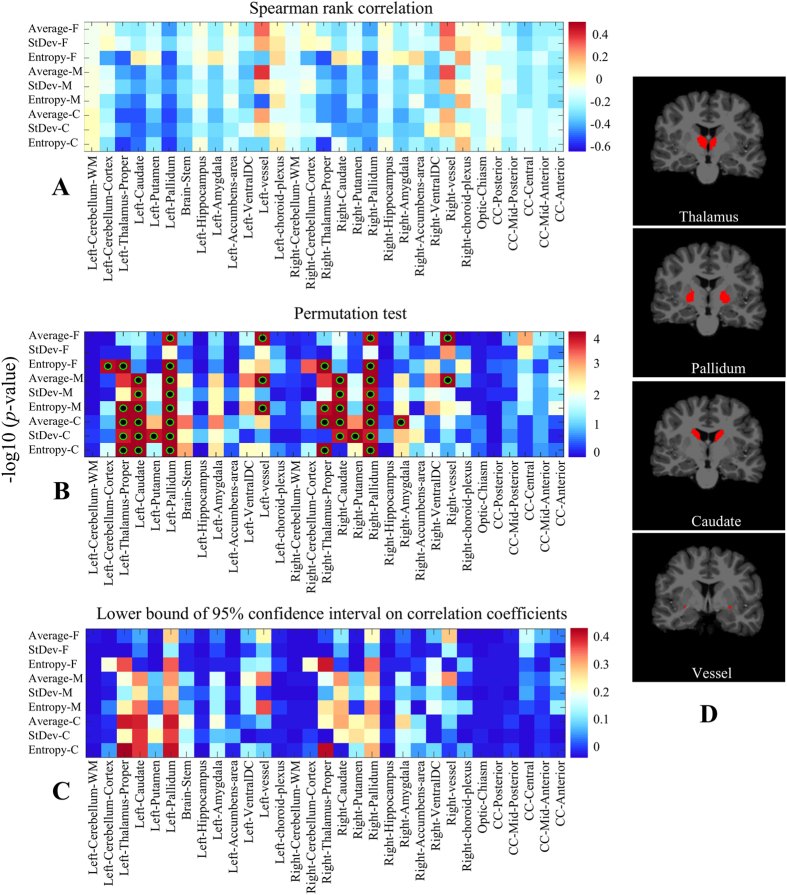
Region-wise correlation between texture features and age in TDC subjects. (**A**) Heatmap of Spearman rank correlation coefficients. (**B**) Heatmap of uncorrected *p*-values (−log_10_ space), based on 100,000 permutations of age labels. Green-black circles indicate region-scale values with significant *p*-values following Holm-Bonferroni correction. (**C**) Lower bound of the 95% confidence interval of absolute correlation coefficients, based on 100 bootstrap samples of 100 TDC subjects. (**D**) Regions showing significant (moderate or high) correlation between texture and age, with corrected *p* < 0.05.

**Figure 8:**
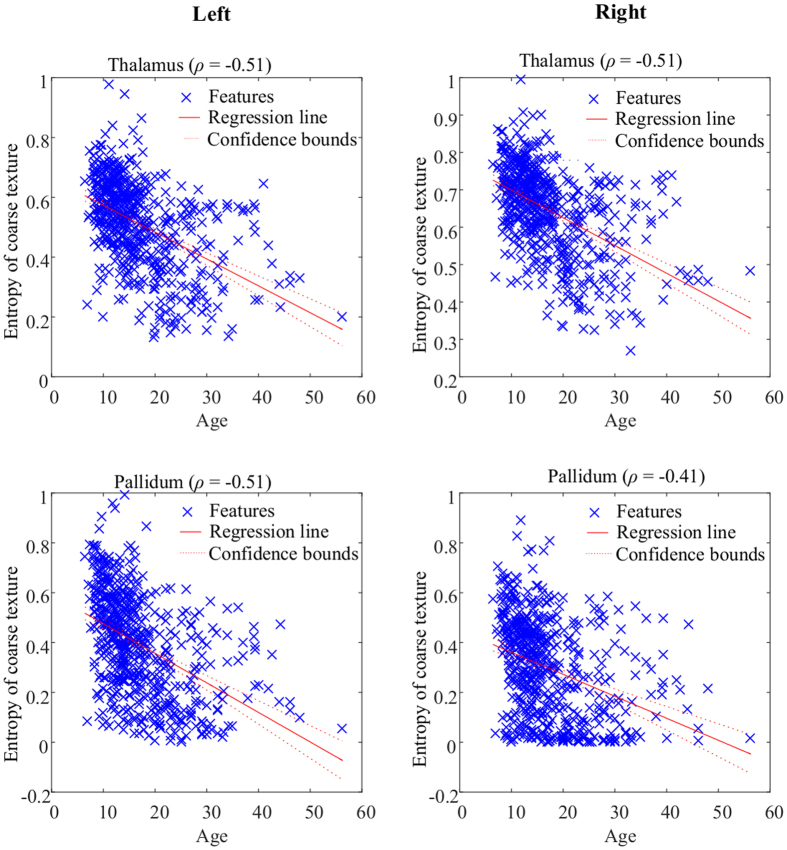
Scatter plots of the Entropy (coarse texture) feature derived from thalamus and pallidum versus age, with Spearman rank correlation coefficients (*ρ*) shown above each panel. Note the degree of bilateral symmetry.

The Spearman rank correlation coefficients between the texture features extracted in each brain region and the age of all 573 healthy control subjects are presented in Fig. 7A and Table 4. These results show a moderate negative correlation (i.e. −0.3 to −0.5) in the thalamus, caudate and pallidum regions, as well as a moderate positive moderate correlation (i.e., 0.3 to 0.5) in the vessel region. Moreover, a high negative correlation (i.e., above 0.5) is observed for the coarse entropy feature of the thalamus and pallidum regions. Figure 8 shows a scatter plot of texture entropy vs. age, for the thalamus and pallidum regions. Unlike in the case of autism, a general pattern of bilateral symmetry is observed, suggesting similar correlations between regional textures and age in both right and left hemispheres.

Figure 7B provides the uncorrected *p*-values heatmap obtained from the permutation test. These results confirm the statistical significance of correlated regions in Table 4, i.e. the hypothesis of no correlation can be rejected with a confidence level of 0.05. The relationship between the regions’ texture and age is also supported by the results of Fig. 7C, which shows the lower bound of the 95% confidence interval on correlation values, computed via the bootstrap analysis.

## Discussion

We extracted multi-scale texture features from 31 prominent sub-cortical brain regions, and performed a statistical analysis to identify significant region-wise texture differences between healthy (TDC) and autism spectral disorder (ASD) subjects. Similar analyses were also conducted to identify regions exhibiting sex-related texture differences between males and age-related texture correlations in healthy subjects.

For the autism-related analysis, we identified five regions exhibiting significant textural differences between ASD and TDC groups at a significance level of corrected *p* < 0.05, namely the left and right cerebellar white matter, right hippocampus, left choroid-plexus, and posterior corpus callosum. The amygdala and pallidum also exhibit differences, although these regions are not significant following Holm-Bonferroni correction. These results are generally consistent with previous studies on autism, showing statistically significant volume differences in the cerebellum^6^,^31^, hippocampus^8^, amygdala^10^, and corpus callosum^11^. Differences observed in the cerebellum could also be linked to the higher curvature and sharpened folds of cerebral white matter exhibited in ASD^69^. Although volumetric differences have previously been reported for the putamen^5^, we did not observe significant texture differences for this region. To our knowledge, the choroid-plexus has not been linked to autism so far in the literature. However, ASD may be connected to excessive subarachnoid cerebrospinal fluid (CSF), which is mainly produced in this brain region^70^.

**Figure 9:**
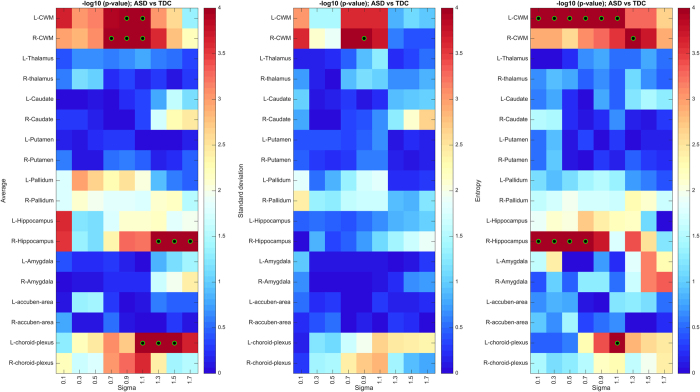
Significance of ASD vs TDC (uncorrected *p*-values, −log10 scale) in selected regions, for various filter scales (sigma). Green-black circles indicate region-scale values with significant *p*-values following Holm-Bonferroni correction.

The analysis of autism-related differences has also underlined the importance of using a multi-scale approach while comparing features in different brain regions. We investigate the effect of filter scale by computing *p*-values for regional feature significance across a dense sampling of the LoG scale parameter (sigma) using the same procedure as in Fig. 5. The result is shown in Fig. 9, where the scale at which peak significance values are observed varies according to the region as well as the quantifier function (i.e., average, standard deviation or entropy). Similarly, in Fig. 11, we achieve similar significance results (i.e. right CWM and hippocampus) after excluding additional patients (i.e. 52 ABIDE volumes flagged as QA failures).

Note that the LoG filter response is strongest when the scale parameter matches the spatial extent of local heterogeneity patterns in the image. Observe also that differential filters such as the LoG operator integrate image information across a finite spatial extent as specified by the scale parameter, and filter responses along region boundaries generally combine intensity information from multiple neuroanatomical regions. Thus although texture features are due primarily to region-specific image content, they may generally include content from neighbouring regions. For instance, while the choroid plexus is a relatively small and narrow structure (see Fig. 5), group differences for this structure are more prominent for larger filter scales. This suggests that filter responses from surrounding regions such as the CSF may explain part of these differences.

Finally, our results pertaining to ASD show average and entropy to be well suited to identifying texture differences in various regions and scales. While both standard deviation and entropy measure feature heterogeneity, which is linked to tissue microstructures, entropy is potentially robust than standard deviation to outlier filter responses, e.g. along tissue boundaries.

For sex-related texture differences in TDC subjects, we identified three regions which exhibit significance at the corrected *p* < 0.05: left amygdala, right cerebellar white matter and brain-stem. Mild differences were also found for the vessel, accumbens area, hippocampus, and corpus callosum (mid anterior sub-region) regions, which are significant prior to correction. While we used a very different methodology, our results on the amygdala may be linked to previous studies that showed a varying pattern of neural connectivity between males and females in this region^71^, as well as sex-related differences in amygdala activity during emotionally influenced memory storage^72^. Likewise, results on the vessel region could be related to previous findings in the literature, showing that cerebral arteries in female brains are significantly less responsive to certain vasoconstrictors than for males^73^. Previous studies have also shown sex differences associated with the hippocampus^74^ and corpus callosum^75^, in particular with respect to brain aging. However, this is the first study to investigate sex-related texture differences in these regions.

**Figure 10:**
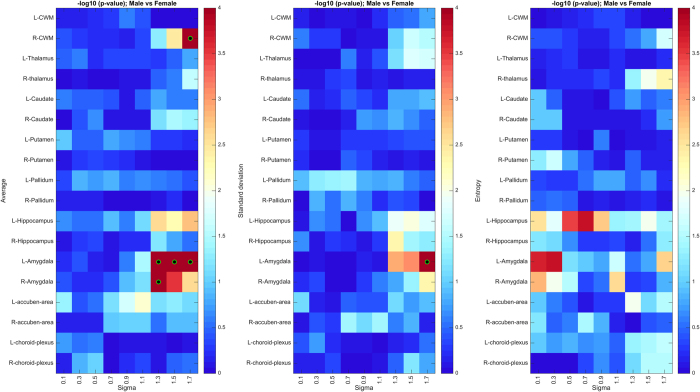
Significance of male vs female (*p*-values, −log10 scale) in selected regions, for various filter scales (sigma). Green-black circles indicate region-scale values with significant p-values following Holm-Bonferroni correction.

**Figure 11:**
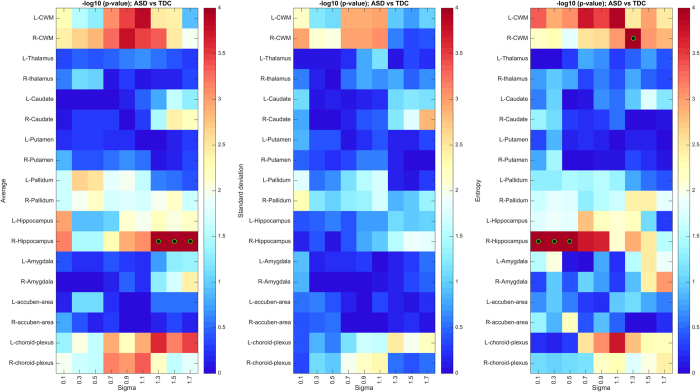
Significance of ASD vs TDC (*p*-values, −log10 scale) in selected regions, for various filter scales (sigma), following exclusion of 52 volumes based on ABIDE QA. Green-black circles indicate region-scale values with significant *p*-values following Holm-Bonferroni correction.

**Figure 12:**
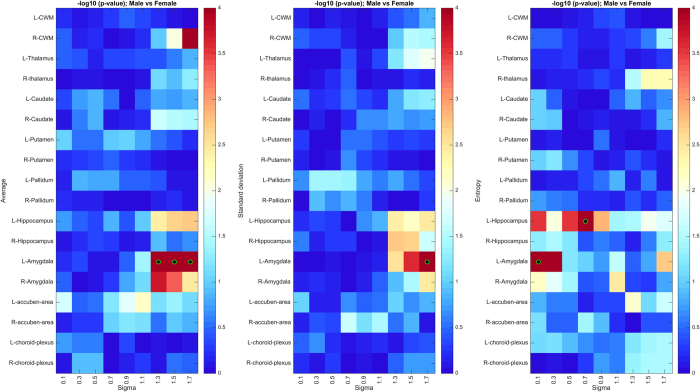
Significance of male vs female (uncorrected *p*-values, −log10 scale) in selected regions, for various filter scales (sigma), following exclusion of 52 volumes based on ABIDE QA. Green-black circles indicate region-scale values with significant *p*-values following Holm-Bonferroni correction.

The analysis of sex-related texture differences also indicates that these differences occur mostly at a coarse texture scale and are more pronounced in the left hemisphere. This can be observed in Fig. 10, showing the significance of male vs. female differences in selected regions across a dense sampling of sigma values. Again, results for sex differences, computed following exclusion of 52 subjects flagged as QA failures in the ABIDE dataset, are highly similar in terms of peak significance values (see Fig. 12). This observation may suggest that differences between male and female brains are mostly structural, and not based on fine-grained tissue properties. Moreover, such differences could be linked to the lateralization of function in brain structures such as the accumbens area and hippocampus. For instance, brain activation in males is lateralized to the left inferior frontal gyrus regions while the pattern of activation is very different in female, engaging more diffuse neural systems that involve both the left and right inferior frontal gyrus^76^. Another study showed that the right hippocampus is believed to be responsible of encoding spatial relationships, while the left has the altered function of storing relationships between linguistic entities^77^. Moreover, females have been shown to make greater bilateral use of the posterior temporal lobes than males during linguistic processing of global structures in a narrative^78^.

Finally, our analysis of age-related differences reveals moderate to high correlations (with corrected *p* < 0.05) between the subjects’ age and textures in the thalamus, caudate, pallidum and vessel regions. These results are consistent with previous studies on the anatomy of aging brains. For instance, changes in thalamus shape and volume have been associated with age^79^, and the volume of the caudate region has been linked to associative memory decline^80^. Another study found a negative correlation between age and the volume of the caudate nucleus, pallidum, amygdala and hippocampus^81^. While our study has shown a link between age and the texture of various brain regions, further investigation is required to fully understand the physiological process responsible for this connection.

In summary, our analysis has highlighted significant texture differences between ASD and TDC, sex differences, and age correlations in key sub-cortical regions. Several results of this analysis are consistent with previous neuroanatomical studies pertaining to autism, sex and age. However, this analysis has also found previously unknown connections between specific regions and autism, sex and age, which offer avenues of further exploration.

Finally, our study has several limitations that merit discussion. First, texture features may be more sensitive to the imaging protocol, noise, and post-processing steps than simple morphological features like volume. In particular, segmentation errors in small and hard to delineate regions could explain some of the significance observed in these regions. Segmentation accuracy is generally worse a priori for regions that are either small or thin, or that exhibit weak intensity contrast along their boundaries. In this work, we used the atlas-based segmentation method of FreeSurfer. While no large-scale study of segmentation accuracy exists for this method, to our knowledge, recent work on sub-cortical parcellation using manually-labelled data have found it to be relatively accurate, with an average Dice coefficient of 78.8% (highest accuracy of 86% in the thalamus, and lowest of accuracy 71% in the pallidum)^82^. Studies have also shown FreeSurfer’s registration and segmentation steps to be robust to age-associated bias^83^, a useful property for our work which uses the data of both preteen and adult subjects. The impact of segmentation errors is also mitigated by the large cohort of subjects used in our study (over 1000) and by our permutation analysis, which offers robust estimates of *p*-values considering the variance introduced by such errors.

Another potential confound is the small number of female subjects in the ABIDE dataset, due to the higher incidence of autism in male subjects. This sex imbalance may introduce a bias when comparing textures across the entire population of subjects. Although we considered this factor by performing permutations tests and deriving confidence intervals via a bootstrap analysis, additional steps could further validate our results. For example, our results could be cross-validated using a different database of subjects, such as the ABIDE II dataset (http://fcon_1000.projects.nitrc.org/indi/abide/abide_II.html). Note that this dataset, containing over 1000 additional subjects (both control and ASD), was not used in our study since a preprocessed version of this database is not currently available. In future work, we plan on extending our study by applying the same preprocessing pipeline to this additional database. Another limitation of our study is the lack of longitudinal data, which would allow us to discover neurodevelopmental patterns related to texture. The ABIDE II dataset contains longitudinal data related to autism, and could be used for such analysis.

## Conclusions

We proposed a region-wise analysis of the brain using multi-scale texture features, and demonstrated its use in identifying regional differences between subject groups (i.e. ASD vs. TDC, male vs. female) and correlations with continuous variables (i.e. age). Findings are generally consistent with results in the literature, with several novel group-related regions identified. To our knowledge, this is the first reported analysis of autism based on MRI texture features.

We conclude that the texture features extracted from hippocampus and cerebellar white matter regions exhibit the most significant differences between ASD and TDC subjects. The choroid-plexus is also identified, to our knowledge this is the first study reporting a potential link between the choroid-plexus and ASD. The significant difference of texture feature between healthy male and female subjects is identified in the amygdala, brain stem and cerebellar white matter regions. Finally, moderate correlations between texture features and the age of healthy subjects are identified in the thalamus, caudate, and pallidum, which are also significant following Holm-Bonferroni correction for multiple comparisons.

These findings suggest that texture features can be a useful representation for characterizing differences in brain structure, that is complementary to traditional voxel-wise or volumetric analysis. Moreover, since texture features can be acquired from arbitrary imaging modalities, the usefulness of our texture-based analysis approach could also tested on modalities other than T1-WI, including other MR modalities or CT scans.

## Additional Information

**How to cite this article**: Chaddad, A. *et al*. Multi-scale radiomic analysis of sub-cortical regions in MRI related to autism, gender and age. *Sci. Rep.* **7**, 45639; doi: 10.1038/srep45639 (2017).

**Publisher's note:** Springer Nature remains neutral with regard to jurisdictional claims in published maps and institutional affiliations.

## Figures and Tables

**Table 1:** Demographic and clinical characteristics of the study groups.

All subjects					TDC groups		
Group	*n*	Male	Female	Age (Average ± StDev)	Group	*n*	Age (Average ± StDev)
ASD	539	474	65	17.01 ± 8.36	Male	474	17.42 ± 7.9
TDC	573	474	99	17.08 ± 7.72	Female	99	15.43 ± 6.56

**Table 2:** Summary of corrected permutation test *p*-values for regions with highest texture differences between ASD and TDC subjects.

Texture scale		Fine			Medium			Coarse		
Brain regions		Average	StDev	Entropy	Average	StDev	Entropy	Average	StDev	Entropy
Left	Cerebellum-WM	0.278	1	0.027*	0.006*	0.067	0.008*	1	1	0.176
	Choroid-plexus	1	1	1	0.059	0.522	0.030*	0.014*	0.979	0.566
Right	Cerebellum-WM	0.761	1	0.540	0.014*	0.014*	0.024*	1	1	0.077
	Hippocampus	1	1	0.014*	0.321	1	1	0.006*	1	1
	CC Posterior	1	1	1	1	1	1	0.498	1	0.04*

Significant features (*p* < 0.05) are represented by a ‘*’ symbol.

**Table 3:** Summary of corrected permutation test *p*-values for regions with highest texture differences between male and female TDC subjects.

Texture scale		Fine			Medium			Coarse		
Brain regions		Average	StDev	Entropy	Average	StDev	Entropy	Average	StDev	Entropy
Left	Amygdala	1	1	0.394	1	1	1	0.006*	0.008*	1
Right	Cerebellum-WM	1	1	1	1	1	1	0.047*	1	1
	Brain-Stem	1	1	1	1	1	1	1	0.022*	1

Significant features (*p* < 0.05) are represented by a ‘*’ symbol.

**Table 4:** Summary of Spearman rank correlation coefficients and corrected permutation test *p*-values (in parenthesis) for regions with the highest correlation between texture and age in TDC subjects.

	Texture scale	Fine			Medium			Coarse		
	Brain regions	Average	StDev	Entropy	Average	StDev	Entropy	Average	StDev	Entropy
Left	Thalamus	−0.20 (1)	−0.04 (1)	**−0.47** (0.003)*	−0.36 (0.068)	−0.27 (1)	−0.39 (0.022)*	**−0.48** (0.005)*	−0.38 (0.043)*	**−0.51** (0.003)*
	Caudate	−0.21 (1)	−0.18 (1)	0.12 (1)	**−0.42** (0.005)*	−0.37 (0.043)*	**−0.43** (0.008)*	**−0.47** (0.003)*	**−0.46** (0.005)*	**−0.45** (0.003)*
	Pallidum	−0.39 (0.041)*	−0.26 (1)	**−0.47** (0.003)*	**−0.43** (0.005)*	−0.39 (0.015)*	**−0.45** (0.005)*	**−0.50** (0.003)*	**−0.46** (0.003)*	**−0.51** (0.003)*
	Vessel	0.38 (0.030)*	0.26 (1)	−0.30 (0.51)	**0.45** (0.003)*	0.15 (1)	**−0.46** (0.003)*	0.29 (0.679)	0.14 (1)	−0.26 (1)
Right	Thalamus	−0.18 (1)	0.03 (1)	**−0.49** (0.003)*	−0.35 (0.066)	−0.25 (1)	−0.38 (0.034)*	−0.39 (0.010)*	−0.06 (1)	**−0.51** (0.003)*
	Caudate	−0.25 (1)	−0.22 (1)	0.16 (1)	**−0.42** (0.008)*	−0.37 (0.036)*	**−0.42** (0.010)*	**−0.42** (0.003)*	−0.36 (0.047)*	−0.04 (1)
	Pallidum	−0.37 (0.041)*	−0.25 (1)	**−0.45** (0.003)*	**−0.41** (0.015)*	−0.36 (0.041)*	**−0.43** (0.005)*	−0.39 (0.013)*	−0.36 (0.045)*	**−0.41** (0.015)*
	Vessel	**0.40** (0.017)*	0.32 (0.221)	−0.12 (1)	**0.42** (0.003)*	0.25 (1)	−0.27 (1)	0.25 (1)	0.19 (1)	−0.15 (1)

Significant features (*p* < 0.05) are represented by a ‘*’ symbol. Correlation coefficients with absolute *ρ* > 0.4 are highlighted using bold font.
